# Transcriptome Profiling of Leaf Elongation Zone under Drought in Contrasting Rice Cultivars

**DOI:** 10.1371/journal.pone.0054537

**Published:** 2013-01-23

**Authors:** Andrew J. Cal, Dongcheng Liu, Ramil Mauleon, Yue-Ie Caroline Hsing, Rachid Serraj

**Affiliations:** 1 International Rice Research Institute, Los Baños, Philippines; 2 Institute of Plant and Microbial Biology, Academia Sinica, Taipei, Taiwan; The Australian National University, Australia

## Abstract

Inhibition of leaf elongation and expansion is one of the earliest responses of rice to water deficit. Despite this sensitivity, a great deal of genetic variation exists in the extant of leaf elongation rate (LER) reduction in response to declining soil moisture. We analyzed global gene expression in the leaf elongation zone under drought in two rice cultivars with disparate LER sensitivities to water stress. We found little overlap in gene regulation between the two varieties under moderate drought; however, the transcriptional response to severe drought was more conserved. In response to moderate drought, we found several genes related to secondary cell wall deposition that were down regulated in Moroberekan, an LER tolerant variety, but up-regulated in LER sensitive variety IR64.

## Introduction

The inhibition of leaf expansion under drought is an important adaptive mechanism to limit leaf area and consequently transpirative loss while water is scarce. Leaf elongation rate (LER) is one of the first physiological parameters to respond to decreasing soil moisture; a reduction in LER precedes changes in transpiration and leaf water potential [1]. Compared with maize or soybean, LER in rice is especially sensitive to drying soil [2]. Although reducing LER can be an important adaptive mechanism for dehydration tolerance, in crop production systems the loss of leaf area during canopy establishment can be disadvantageous for yield, particularly in rain-fed rice ecosystems prone to episodic vegetative drought [3].

Leaf expansion rates are determined by turgor and cell wall extensibility. Despite the sensitivity of rice to drought, some varieties maintain leaf water potential under water deficit through the regulation of stomatal closure. Though a proportion of the genotypic differences in leaf elongation in rice can be attributed to root variability, significant differences in the response of leaf elongation to drought exist even after rooting effects are neutralized [4].

The purpose of this study was to characterize how the transcriptome response to water deficit of the rice leaf elongation zone differs between contrasting varieties. We have measured global gene expression of the leaf elongation zone in IR64, a drought-sensitive modern indica variety, and Moroberekan, a drought-tolerant tropical japonica landrace [5]. Both genotypes were grown in common bins using the fraction of transpirable soil water as a drought co-variable to minimize differences in transpiration and root system architecture. The fraction of transpirable soil water (FTSW) protocol [6] was used for stress imposition; leaf elongation and transcript levels were measured coincidentally under well-watered (FTSW 1), moderate drought stress (FTSW 0.5), and severe drought stress (FTSW 0.2) conditions.

## Results and Discussion

### Genotypic Response of Leaf Elongation to Soil Drying

**Figure 1:**
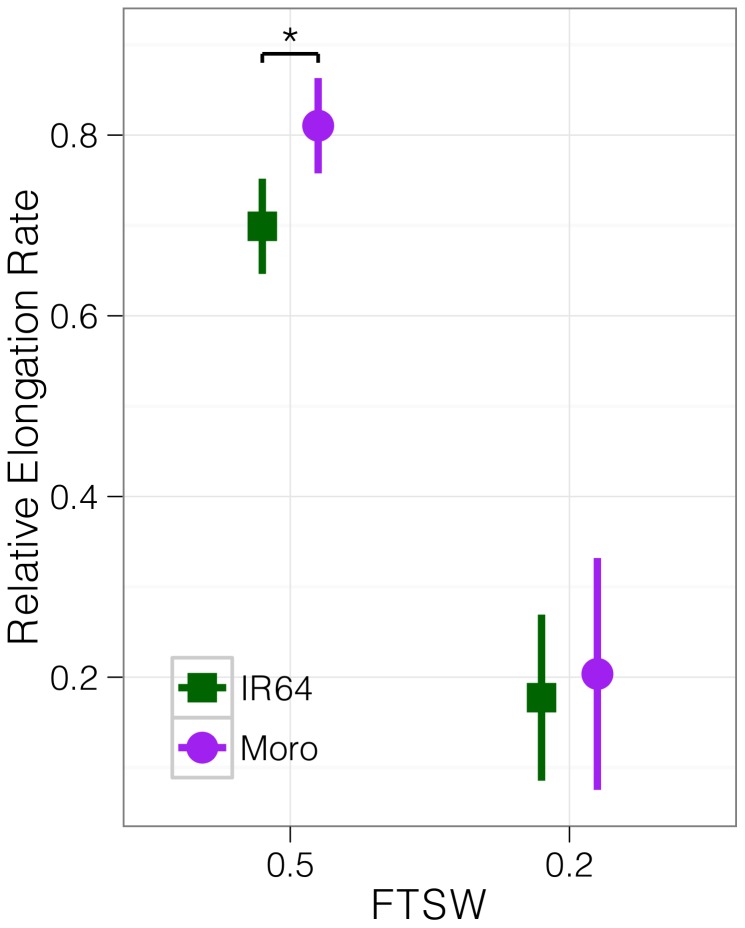
Relative leaf elongation under soil drying. Bars represent +/- SE.

In order to investigate genotypic response to drought, we measured leaf elongation concurrently under well-watered (FTSW 1), moderate soil drying (FTSW 0.5), and severe soil drying (FTSW 0.2) conditions. Leaf elongation rates were greater in Moroberekan than in IR64 under all water regimes (Figure 1). The reduction of leaf elongation rates in response to drought relative to irrigated conditions was significantly lower in Moroberekan than in IR64 at FTSW 0.5 (t-test p = 0.006) but not at FTSW 0.2 (p = 0.24). Our results indicate that genotypic differentiation of leaf elongation rates is greater under moderate drought stress, whereas under severe stress elongation rates decline sharply in both genotypes.

Importantly, this result indicates that genotypic comparisons of growth response dynamics to water deficit are more relevant under mild stress than under severe drought, when most of the cellular and metabolic processes are affected and the plant reaches drought survival phase [7]. We failed to find any differences in leaf water potential between treatments or genotypes, consistent with previous research characterizing rice leaves as isohydric [4].

### Transcriptome Response of Leaf Elongation Zone to Soil Drying

**Figure 2:**
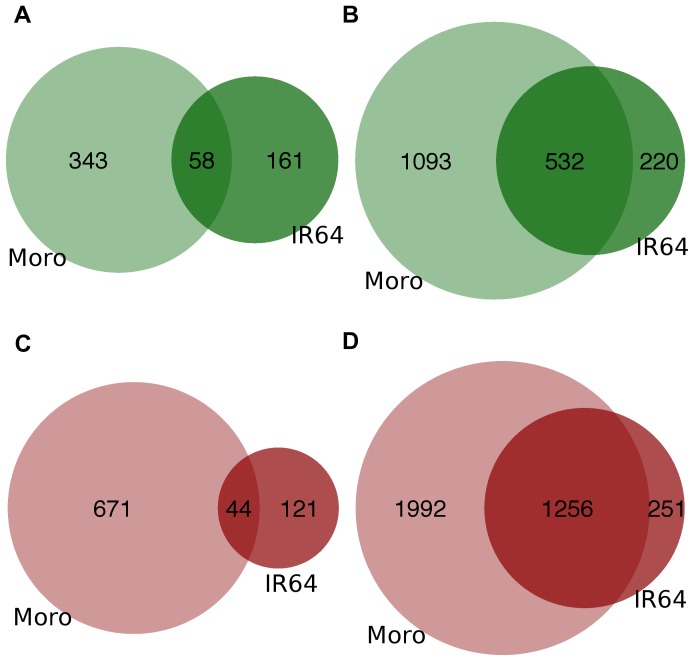
Overlap of DEGs between IR64 and Moroberekan. A. Up-regulated FTSW 1-0.5 B. Up-regulated FTSW 0.5-0.2 C. Down-regulated FTSW 1-0.5 D.Down-regulated FTSW 0.5-0.2. The area of circles and their overlap is proportional to the number of genes represented.

In order to understand the molecular events underlying phenotypic differences in LER, we measured gene expression in the leaf elongation zone of Moroberekan and IR64 under well-watered, moderate soil drying, and severe soil drying conditions (FTSW 1, 0.5, and 0.2) using the Affymetrix 57 k rice array. The extent of transcriptome remodeling under severe stress was much greater than under moderate stress: greater than ten times more genes were differentially expressed between moderate and severe stress than between well-watered and moderate stress (Table 1). At a false discovery rate (FDR) of 0.1, 58 genes were up-regulated and 47 genes were down-regulated in both IR64 and Moroberekan from FTSW 1-0.5 (Figure 2).

**Table 1:** Number of differentially expressed genes for each condition at FDR 0.1 and 0.01.

FDR		FTSW 1-0.5		FTSW 0.5-0.2		FTSW 1-0.2	
		up	down	up	down	up	down
IR64	0.1	219	165	752	1507	1642	3544
	0.01	75	56	373	634	907	1948
Morob.	0.1	401	715	1625	3254	2280	3586
	0.01	143	259	895	1846	1429	2142

### Transcriptional Differences between Genotypes are the more Numerous than Drought-induced Transcriptional Changes

**Figure 3:**
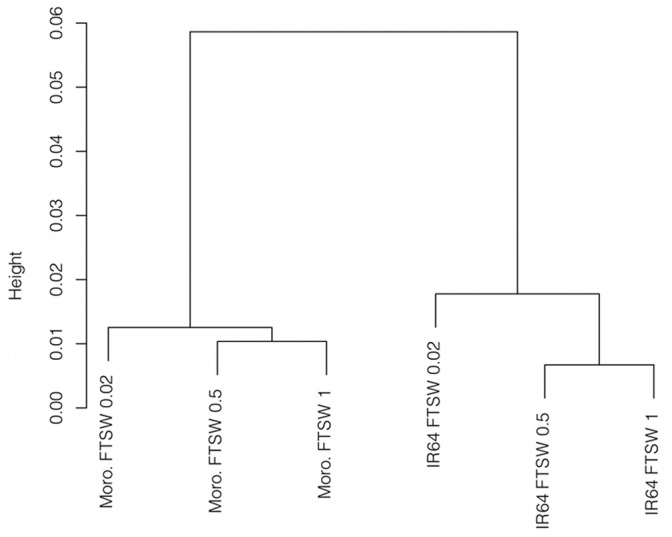
Hierarchical clustering of genotype/treatment combinations based on normalized expression of 23,945 genes.

Hierarchical clustering revealed a deep divide in expression patterns between genotypes (Figure 3). The severe stress treatment clustered away from moderate and irrigated conditions in both genotype groups.

Over two thousand expression level polymorphisms were detected between genotypes under irrigated conditions at FDR 0.01 (Table 2). Expression level polymorphisms increased around 40% under both moderate and severe drought relative to well-watered conditions (Table 2).

**Table 2:** Expression level polymorphisms for each condition at FDR 0.1 and 0.01.

	FTSW 1		FTSW 0.5		FTSW	
	IR. > Mor.	Mor. > IR.	IR.>Mor.	Mor. > IR.	IR. > Mor.	Mor. > IR.
FDR 0.1	1650	1540	2389	1906	2567	1898
FDR 0.01	1057	1185	1701	1414	1667	1419

### Clock Genes are Down-regulated Under Moderate Soil Drying

Among common genes down-regulated from FTSW 1-0.5, the gene ontology category “rhythmic process” was found to be enriched. Differentially expressed genes (DEGs) in this category include OsGigantea (OsGI) and Os11g34460, the rice ortholog of FKF1, which together act to induce *Constans* and promote flowering in *Arabidopsis* [8]. *Constans* is an ortholog of Hd1, a major determinant of flowering time in rice, and it promotes flowering under short day conditions [9]–[10]. Delayed flowering is a common response of rice under vegetative drought stress [11]–[12], and our observed down-regulation of early pathway components OsGI and OsFKF1 may be part of the underlying molecular basis for this phenomenon. Other clock-associated genes that were commonly down-regulated from FTSW 1-0.5 include psuedo-response regulators OsTOC1 and OsPRR95. In *Arabidopsis*, TOC1 RNAi lines show increased survival to dehydration and greater stomatal closure, and the gene has been been implicated in gating plant sensitivity to ABA [13]. Although we did not observe any changes in ABA biosynthetic enzymes, several ABA-responsive transcription factors were up-regulated under mild stress, including heat shock factors Os09g35790 and Os10g28340. Although these genes could be responding to increased ABA produced in other tissues, our results suggest that clock perturbation may be affecting ABA response in the rice leaf elongation zone. No gene ontology categories were overrepresented among genes up-regulated in both varieties in response to mild stress.

### Central Metabolic Pathways are Down-regulated Under Severe Soil Drying

Down-regulation was predominant under severe stress; we found more than twice as many genes commonly down-regulated as were up-regulated in these contrasts (Table 1). Our analysis of gene ontology (GO) categories revealed significant enrichment for down-regulation of central cellular metabolism, such as “translation”, “glycolysis”, and “porphyrin biosynthetic process” (Table S1). Among up-regulated genes, we found enrichment for GO categories “protein amino acid dephosphorylation”, “response to water”, and “chaperone mediated protein folding”. This transcriptional re-programming reflects a down-regulation of core metabolic processes in response to dehydration, consistent with stage III or the survival stage of drought stress when nearly all available soil moisture has been exhausted [6]. Down-regulation of categories such as “response to water” is similar to findings of previous microarray studies [14]. Additionally, several GO categories related to H+-ATPases were also enriched in down-regulated DEGs (Table S1). Acidification of the extracellular matrix is an important process for cell wall expansion, and this expression shift could play a role in the decrease in leaf expansion during the transition from moderate to severe stress [15]–[16].

### Genotypic Differentiation of Transcriptome Response is Greatest Under Moderate Soil Drying

We observed little overlap between IR64 and Moroberekan in DEGs from FTSW 1-0.5. Only 5% of down-regulated DEGs and 10% of up-regulated DEGs in this contrast are differentially expressed in both genotypes. The overlap of DEGs from FTSW 1-0.2 and FTSW 0.5-0.2 is several times greater: 40% and 27% of all genes down/up DEGs between FTSW 1 and 0.2 were common between IR64 and Moroberekan. Across contrasts and FDRs, we found approximately twice as many genes differentially regulated in Moroberekan, a drought tolerant tropical japonica, than in IR64, a drought-susceptible improved indica [5]. Our results are similar to the results of a previous study that found far more genes regulated under drought in more tolerant osmotic-adjusting lines than in low-adjusting lines [16].

**Figure 4:**
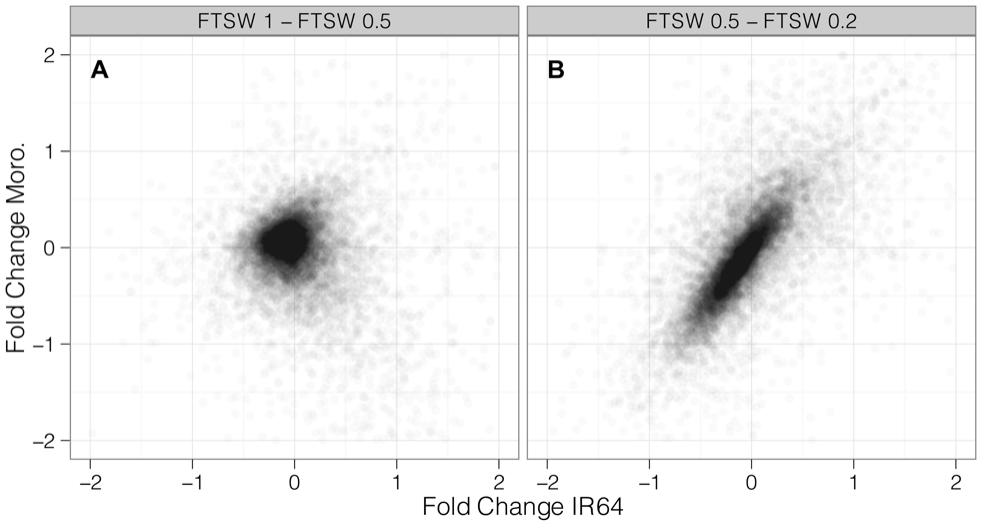
Genotypic differentiation of transcriptome response to moderate and severe soil drying. Fold change of all genes for IR64 (x- axis) and Moroberekan (y-axis) from FTSW 1-0.5 (panel A, r^2^ = −0.22) and FTSW 0.5-0.2 (panel B, r^2 = ^0.63).

We found greater genotypic differentiation in transcriptome response during early drought stress than to severe stress (Figure 4). Comparing the fold change for all genes between IR64 and Moroberekan from FTSW 1-0.5, we found a slight negative correlation between genotypes (r^2^ = −0.22, p < 2.2E-16), whereas we observed a much stronger, positive correlation of fold change between genotypes from FTSW 0.5-0.2 (r^2^ = 0.63, p < 2.2E-16). These results indicate that the molecular response to severe soil drying is conserved between a drought-susceptible, high yielding indica and tolerant tropical japonica. Our results suggest that the early stages of soil drying may be critical to understanding genotypic response to drought.

To understand genotypic differences in gene regulation from well-watered conditions to mild stress, we looked for genes that were differentially expressed in both genotypes but that had opposite patterns of regulation. At FDR 0.1, we found 26 genes that were changed in opposite directions between genotypes from FTSW 1-0.5. Interestingly, 25 of these genes exhibited an increase in expression in IR64 and a decrease in Moroberekan. We found this set of genes significantly enriched for the GO cellular component “cell wall”.

### Differential Regulation of Cell Wall Genes between Genotypes Under Moderate Stress

**Figure 5:**
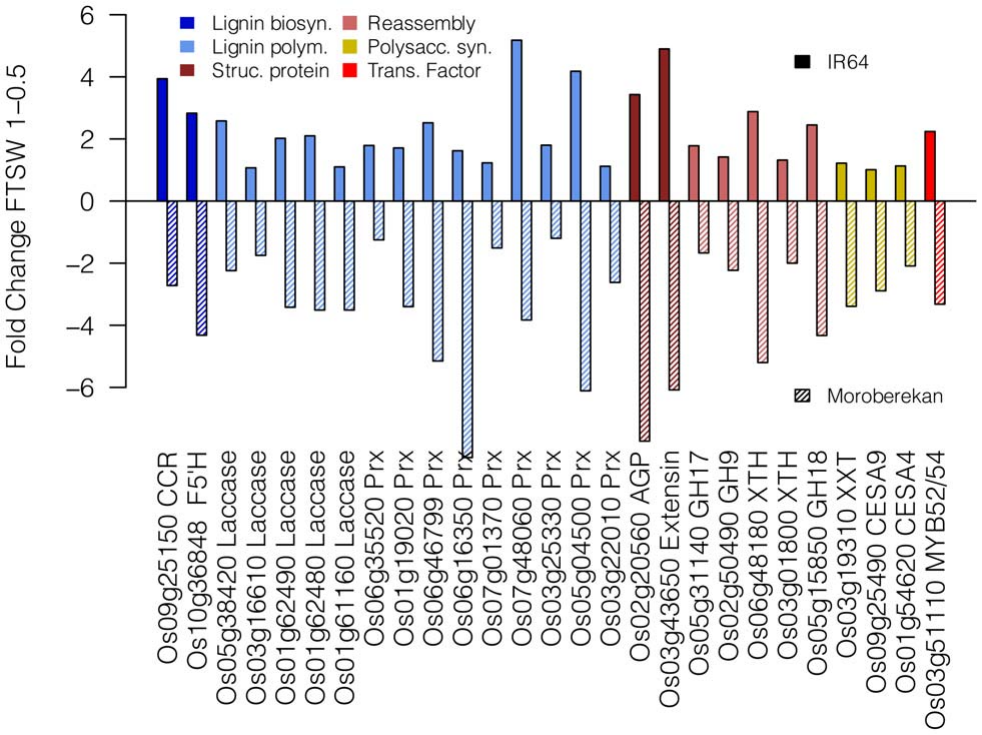
Cell Wall DEGs. Fold change difference for all cell wall annotated genes with at least two-fold change under mild stress in both IR64 (solid bars) and Moroberekan (striped bars).

In order to determine which cell wall genes were differentially regulated in IR64 and Moroberekan from FTSW 1-0.5, we examined gene families involved in cell wall deposition and structure for genes with greater than a two-fold expression change in both genotypes [17]–[19]. Surprisingly, all the genes we identified under these criteria were oppositely regulated between genotypes with the same directionality: the expression of these genes under moderate water deficit increased in IR64 and decreased in Moroberekan (Figure 5). Of the 27 genes we identified through this analysis, 8 also reached transcriptome-wide significance in both genotypes at FDR 0.1. The largest number of genes were involved in secondary cell wall deposition. This set featured both genes participating in monolingol biosynthesis, such as a cinnamoyl-CoA reductase and ferulate-5-hydroxylase, and genes associated with lignin polymerization, including five laccases and ten apoplastic class III peroxidases. Increased lignification has been observed in the leaf elongation zone of droughted maize leaves [20]. Increased cell wall peroxidase activity has been implicated in the cessation of leaf elongation during normal development [21] and under drought [22].

Cell wall structural proteins were also found to be alternately regulated with large fold-changes under mild drought stress, including an arabinogalactan protein and LRR-extensin. Extensins are cross-linked by class II peroxidases as well, resulting in less extensible cell walls [23]. Several glycosyl hydrolases were differentially expressed under drought, including two xyloglucan endotransglucosylase/hydrolases (XTHs). XTH genes are known to be involved in cell loosening and elongation [24], [25], but may also have a role in cell wall strengthening [26], particularly in the relatively pectin-poor cell walls of the *Poacea* [27]. Finally, three glycosly transferases were alternately regulated, including two cellulose synthase A subunits.

The only two cell wall DEGs up-regulated in Moroberekan under mild stress, Os01g66710 and Osg05g20020, both belong to glycosly hydrolase family GH28. This family of polygalacturonases has been found to be up-regulated in elongating maize internodes relative to internodes that have ceased elongation [28].

Our observations suggest that these two varieties have alternative strategies for the regulation of leaf elongation under mild soil drying between the two varieties. In IR64, which is adapted to flooded production systems, moderate drought is strongly inhibitory of LER and increases expression of cell wall cross-linking genes. In Moroberekan, which is adapted to upland production systems in which soil moisture fluctuates, we find decreased cell wall gene expression, especially for secondary cell wall genes, with a slower inhibition of LER. An examination of enzymatic activities and cell wall extensibility is needed to confirm these hypotheses.

We also examined transcription factors with known roles in the regulation of cell wall gene expression [29]. Myb52/54, a transcription factor previously shown to be involved in secondary cell wall gene expression [30], was over two-fold down-regulated in Moroberekan and two-fold up-regulated in IR64 under mild soil drying (Figure 5). Another cell wall transcription factor, Myb58/63, was also down-regulated in Moroberekan, though expression was unchanged in IR64. Similar to our observations, down-regulation of these transcription factors has previously been observed to be correlated to the down-regulation of lignin biosynthetic pathways; however, contrary to these previous findings we did not observe a coordinated up-regulation of cellulose biosynthetic genes, indicating that drought may disrupt the normal developmental coordination in cell wall biosynthesis [29]. Further genetic dissection is necessary to identify upstream regulatory events that affect differential cell wall regulation between these genotypes.

### Conclusions

Our studies reveal differences in leaf elongation rates under drought between a drought-sensitive super-variety and a drought-tolerant landrace. Our transcriptome profiling of the leaf elongation zone under drought suggests opposite regulation of cell wall strengthening as a molecular mechanism underlying genotypic differentiation in leaf elongation. The expression profiles under two different levels of soil water deficit demonstrate divergent regulatory regimes under mild and severe stress; mild stress induces a host of regulatory and cell wall expression changes, whereas severe stress leads to the down-regulation of central metabolic processes and preparation of dehydration. Between diverse cultivars, we find that the transcriptional program under severe drought to be much more conserved than under mild drought. Finally, we suggest that transcriptional regulation of clock components may lead to drought induced flowering delay in rice.

## Materials and Methods

### Plant Growth Conditions

Plants were grown in the IRRI phytotron in October/November 2007. Trays (25 cm × 40 cm × 15 cm) were filled with two kg dried, sieved soil. 60 pre-germinated seedlings per tray were sown in pairs; after seven days one seedling per pair was removed, leaving six rows each containing five plants. Genotypes were sown in alternating rows within the same tray. Accessions used for this study were IR64 and Moroberekan (IRIS GID 2254729 and 2254722).

Trays assigned to the drought treatments were drained overnight after the emergence of the 6th leaf and allowed to dry until target FTSW. Trays were weighed and re-watered four times daily to maintain target FTSW. Sampling for RNA was conducted co-incidentally for all treatments at the 7th leaf stage between 9 and 11 AM. Approximately three cm of the leaf elongation zone was excised from elongating leaves; all samples of the same genotype from a given tray were bulked for RNA extraction. Three biological replicates were used for array hybridization; each biological replicate was comprised of leaf elongation zones sampled from different replicate trays.

### Leaf Elongation

Leaf elongation of emerging seventh leaves was measured after FTSW targets were reached. Leaf elongation was measured as the difference in leaf length after 12 and 18 hours and the leaf elongation rate (LER) was computed based on the duration. The measurement was performed on at least five plants for each tray in four trays per treatment.

### RNA Extraction, Labeling, and Array Hybridization

Three biological replicates of the Sampled leaf tissues were ground in liquid nitrogen and RNA was extracted using the TRIZOL reagent (Sigma Chemical Co., USA) according to the manufacturer’s instructions, and crude RNA preparations were treated with DNase (Invitrogen, USA), following extraction. Samples were hybridized to the Affymetrix Rice Genome array (GEO #GPL2025). Preparation of labeled material for array hybridization was carried out according to manufacturer’s instructions (Affymetrix, Santa Clara, CA). Briefly, 2 µg of total RNA was used for synthesizing ds cDNA. Biotin-tagged cRNA was generated from an in vitro transcription reaction using MessageAmp™II aRNA Amplification Kit and then fragmented into 35–200 bases in length. The resulting cRNA was then hybridized to the Affymetrix rice genome array. Hybridization was processed at 45°C, with rotation for 16 h (Affymetrix GeneChip Hybridization Oven 640). The arrays were washed and stained in the Affymetrix Fluidics Station 450 and scanned using the Affymetrix Gene Chip Scanner 3000. The microarray hybridization and scanning were conducted in the DNA microarray core laboratory of the Institute of Plant and Microbial Biology, Academia Sinica (IPMB).

### Microarray Analysis

Array data was analyzed using the PUMA package in R [31]. Arrays were pre-processed and normalized with the mmgmos function in PUMA. The probability of positive likelihood ratio (PPLR) is calculated using a Bayesian hierarchical model in PUMA. This statistic was then converted into “P-like values,” which represent the probability that a given gene is differentially expressed. FDR for transcriptome-wide thresholds was calculated using Benjamini and Hochberg method [32] The Affymetrix cdf for the rice array contains multiple probe sets mapping to the same gene. To eliminate this issue, a transcript-consistent alternate cdf file was generated using Affyprobeminer (http://gauss.dbb.georgetown.edu/liblab/affyprobeminer/ [33]) to NCBI CCDS sequences. A minimum of 5 probes were used per transcript. GoSlim assignments were downloaded from the MSU Rice Genome Annotation Project and full GO assignments were integrated from RAP and Gramene annotations. GO enrichment was evaluated using Fisher’s exact test in R; resulting p-values were adjusted according to the Benjamini and Hochberg method [32].Hierarchical cluster was performed using the hclust function in R. Gene IDs were converted with RAP ID converter (http://rapdb.dna.affrc.go.jp/tools/converter). Cell wall gene family annotations were downloaded from UC Riverside Cell Wall Navigator (http://bioweb.ucr.edu/Cellwall) [17], Gramene RiceCyc (http://www.gramene.org/pathway/ricecyc.html [18] for phenylpropenoid biosynthesis), and Purdue cell wall genomics (http://cellwall.genomics.purdue.edu/families/index.html [19], for laccases and peroxidases).

### Accession Numbers

Microarray data and cdf file have been deposited with the NCBI Gene Expression Omnibus (GSE41159, GPL16106).

## Supporting Information

Table S1 Gene ontology category enrichment of up and down-regulated genes and expression level polymorphisms.
(XLS)
